# Identification of highly reliable risk genes for Alzheimer’s disease through joint-tissue integrative analysis

**DOI:** 10.3389/fnagi.2023.1183119

**Published:** 2023-06-21

**Authors:** Yong Heng Wang, Pan Pan Luo, Ao Yi Geng, Xinwei Li, Tai-Hang Liu, Yi Jie He, Lin Huang, Ya Qin Tang

**Affiliations:** ^1^Department of Bioinformatics, School of Basic Medical Sciences, Chongqing Medical University, Chongqing, China; ^2^Joint International Research Laboratory of Reproduction and Development, Chongqing Medical University, Chongqing, China; ^3^School of Microelectronics and Communication Engineering, Chongqing University, Chongqing, China

**Keywords:** GWAS, TWAS, Mendelian Randomization, eQTL, Alzheimer’s disease

## Abstract

Numerous genetic variants associated with Alzheimer’s disease (AD) have been identified through genome-wide association studies (GWAS), but their interpretation is hindered by the strong linkage disequilibrium (LD) among the variants, making it difficult to identify the causal variants directly. To address this issue, the transcriptome-wide association study (TWAS) was employed to infer the association between gene expression and a trait at the genetic level using expression quantitative trait locus (eQTL) cohorts. In this study, we applied the TWAS theory and utilized the improved Joint-Tissue Imputation (JTI) approach and Mendelian Randomization (MR) framework (MR-JTI) to identify potential AD-associated genes. By integrating LD score, GTEx eQTL data, and GWAS summary statistic data from a large cohort using MR-JTI, a total of 415 AD-associated genes were identified. Then, 2873 differentially expressed genes from 11 AD-related datasets were used for the Fisher test of these AD-associated genes. We finally obtained 36 highly reliable AD-associated genes, including APOC1, CR1, ERBB2, and RIN3. Moreover, the GO and KEGG enrichment analysis revealed that these genes are primarily involved in antigen processing and presentation, amyloid-beta formation, tau protein binding, and response to oxidative stress. The identification of these potential AD-associated genes not only provides insights into the pathogenesis of AD but also offers biomarkers for early diagnosis of the disease.

## Introduction

Alzheimer’s disease (AD) is the most common form of dementia, accounting for approximately 50–60% of dementia cases. As a progressive neurodegenerative disorder, AD is characterized by a gradual loss of memory and cognition, with amyloid plaques and neurofibrillary tangles being the primary pathological features (Hansson et al., 2018). The recent data suggest that there are around 55 million people worldwide living with dementia, and the prevalence of dementia is expected to triple by 2050 (Khan et al., 2020). This increase will impose a significant burden on both families and society, making it crucial to analyze the pathogenic mechanism and identify potential risk factors for AD diagnosis and treatment.

Genome-wide Association Studies (GWAS) have emerged as a powerful tool for investigating complex diseases, leading to the discovery of over 40 AD-associated risk alleles through large cohort studies on AD (Jansen et al., 2019; Scheltens et al., 2021). However, due to the strong linkage disequilibrium (LD) among variants, the loci identified by GWAS cannot be interpreted directly, which further obscures the causality between variants and phenotypes. Therefore, GWAS data alone is insufficient for determining the causal genes and underlying regulatory mechanisms. To fill this gap, transcriptome-wide association studies (TWAS) are valuable developed for studying potential gene regulatory mechanisms associated with variable traits by integrating transcriptomic and genetic data (Tang et al., 2021). Traditional TWAS analysis typically involves three stages: (i) Construction of association between single nucleotide polymorphisms (SNPs) and gene expression using weighted calculations based on reference panels such as the GTEx database or other cohort data containing both genotyping and expression (known as the expression quantitative trait locus or eQTL model). (ii) Filling the lacked gene expression data in the large-scale GWAS cohort using the trained eQTL model. (iii) Utilizing the filled gene expression data to infer associations between gene expression and traits (Luningham et al., 2020). To achieve more accurate results, several improved methods have been developed based on the traditional TWAS approach, including PrediXcan (Gamazon et al., 2015), S-PrediXcan (Barbeira et al., 2018), UTMOST (Rodriguez-Fontenla and Carracedo, 2021), CoMM (Yang et al., 2019), PMR-Egger (Yuan et al., 2020), moPMR-Egger (Liu et al., 2021), VC-TWAS (Tang et al., 2021), TIGAR (Nagpal et al., 2019). TWAS has proven successful in integrating transcriptomic and genetic data to study various complex human diseases, such as schizophrenia, breast cancer, prostate cancer, and Crohn’s disease (Tang et al., 2021).

**FIGURE 1:**
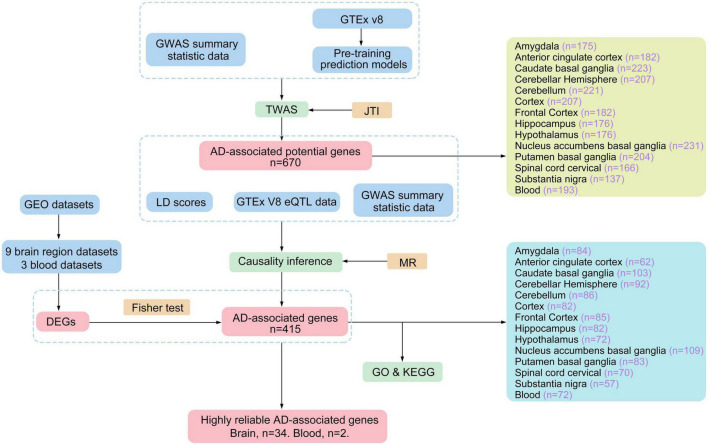
The work flow chart of the experiment.

In 2020, Zhou et al. (2020) presented a new method to optimize TWAS, namely the Joint-Tissue Imputation (JTI) approach and a Mendelian Randomization (MR) framework for causal inference (MR-JTI). Traditional TWAS methods often fail to fully capitalize on the shared biological characteristics across multiple tissues in the GTEx dataset during the training of the prediction model. Consequently, the prediction accuracy decreases. In contrast, JTI effectively leverages the common regulatory architecture of gene expression across multiple tissues. When the transcriptional regulation of the target gene in simple tissue is specific, it will automatically restore the model to a single tissue prediction model, PrediXcan, thus improving tissue specificity in prediction. These advancements make JTI a superior prediction method compared to PrediXcan, BSLMM, TIGAR, and UTMOST models (Zhou et al., 2013). Furthermore, JTI incorporates Mendelian Randomization (MR) into its framework, significantly enhancing the evidence level and credibility of association analysis. This integration helps address false positive issues caused by horizontal gene pleiotropy and potential confounding factors. In this study, we conducted tissue-specific TWAS for AD across 13 brain regions and blood. We utilized GWAS data and eQTL cohorts from GTEx (version 8) and employed the MR-JTI method. The experimental flow chart detailing the process is depicted in Figure 1. Our findings provide more reliable potential biomarkers and targets for investigating the pathogenesis of AD.

## Materials and methods

### Public GWAS statistics data of AD

The GWAS summary statistics data for AD were obtained from the published study in 2022, which included 111,326 AD cases and 677,663 controls (Bellenguez et al., 2022). The data were downloaded from the European Bioinformatics Institute GWAS Catalog^1^ with accession number GCST90027158. For detailed information about the sample collection, analysis methods, and results, please refer to the original publication (Bellenguez et al., 2022).

### GTEx quantitative trait loci (eQTL) data

The GTEx project is the continuously updated public database of human genetic resources. Its latest version V8, contains massive sequencing data of 54 non-diseased tissue sites from 948 donors (Consortium et al., 2017). Using the gene expression and genotype data, the GTEx project performed the eQTL analysis of each tissue site, and the eQTL models can be obtained from its website.^2^ Zhou et al. (2020) developed the joint-tissue imputation (JTI) approach by considering the shared genetic regulation effects across different tissues and the unique genetic regulation in the target tissues. They trained the GTEx eQTL data with JTI to enhance the prediction performance of instrumental variables for genes. The JTI pre-training models was downloadable, including the eQTL summary statistics and the SNP-SNP covariance matrices^3^ (Zhou et al., 2020). Here, we obtained the trained pre-trained prediction models for 13 brain regions and blood, including brain amygdala, brain anterior cingulate cortex, brain caudate basal ganglia, brain cerebellar hemisphere, brain cerebellum, brain cortex, brain frontal cortex, brain hippocampus, brain hypothalamus, brain nucleus accumbens basal ganglia, brain putamen basal ganglia, brain spinal cord cervical, and brain substantia nigra.

### Joint-tissue imputation (JTI) and Mendelian randomization (MR) integrative analysis (MR-JTI)

The TWAS analysis was performed by JTI using the pre-training eQTL models and GWAS summary statistic data. The *P*-values of genes in JTI analysis were adjusted using the p.adjust function in R (version 4.1.3) with the false discovery rate (FDR) method. Genes with FDR < 0.05 were identified as AD-associated genes. While JTI established the relationship between gene expression and AD, it remained unclear whether the differential expression of these genes was the cause or the result of AD. Consequently, the identified AD-associated genes were employed for subsequent MR analysis to elucidate the causal relationship between candidate genes and AD. To mitigate confounding effects, LD scores were calculated. These LD scores were obtained using the GCTA software, utilizing data from the 1000 Genomes Project,^4^ which created a catalog of common human genetic variation by using openly consented samples from people who declared themselves to be healthy. The MR-JTI analysis combined LD scores, GTEx eQTL data, and GWAS summary statistics to obtain potential AD-associated genes.

### Analysis of differentially expressed genes in AD

Gene Expression Omnibus (GEO)^5^ database is a public genomic database containing the entire gene expression data, chips, and microarrays. The 11 published datasets of AD-associated were retrieved and downloaded from the GEO database (Table 1). Differentially expressed genes (DEGs) were identified using a *P* < 0.05 and | log fold-change (FC)| > 1. The gene symbols of datasets were annotated using DAVID online software.^6^ Finally, the AD-associated genes identified by MR-JTI and the AD-associated DEGs were tested by the Fisher test to confirm the notable intersection genes.

**TABLE 1:** Details of AD related GEO datasets.

GEO accession	Public data	Tissues	Control	AD case	References
GSE1297	July 2004	Hippocamp	9	22	Blalock et al., 2004
GSE4757	May 2006	Entorhinal cortex	0	10	Dunckley et al., 2006
GSE16759	January 2010	Parietal lobe tissue	4	4	Nunez-Iglesias et al., 2010
GSE36980	April 2013	Frontal cortex/Temporal cortex/Hippocampus	18/19/10	15/10/8	Hokama et al., 2014
GSE110226	February 2018	Entire lateral ventricular choroid plexus	6	7	Kant et al., 2018; Stopa et al., 2018
GSE118553	July 2019	Entorhinal cortex/Temporal cortex/Frontal cortex/Cerebellum brain region	37/52/40/38	24/31/23/22	Patel et al., 2019
GSE39420	January 2015	Posterior cingulate area	7	7	Antonell et al., 2013
GSE37263	April 2012	Temporal cortex	8	8	Tan et al., 2010
GSE4226	October 2006	Peripheral blood mononuclear cells	14	14	Maes et al., 2007; Maes et al., 2009
GSE4227	January 2009	Peripheral blood mononuclear cells	18	16	Maes et al., 2009; Maes et al., 2010
GSE4229	January 2009	Peripheral blood mononuclear cells	22	18	Maes et al., 2009

### Pathway and functional enrichment analysis of AD-associated genes

The Kyoto encyclopedia of genes and genomes (KEGG)^7^ and Gene Ontology (GO)^8^ were used for the pathway and functional enrichment analysis of AD-associated genes by R package ClusterProfiler. The GO enrichment analysis includes biological pathways (biological process, BP), cellular components (CC), and molecular function (MF). Moreover, the R package Circlize was used to visualize the high reliable genes and the significantly enriched pathways (adjusted *P* < 0.05) in which they were involved. Whether the high reliable genes and these pathways have been confirmed to be associated with immunity and AD were evaluated by manual literature search. The high reliable genes were further compared with the immune genes in innateDB^9^ (Breuer et al., 2013) and ImmPort^10^ (Bhattacharya et al., 2018) database to confirm their relationship with immunity.

## Results

### Identification of 415 AD-associated genes by MR-JTI

To identify risk factors associated with AD, we performed the TWAS analysis using JTI pre-training models and GWAS summary statistic data from AD in 2022 (Zhou et al., 2020; Bellenguez et al., 2022). JTI integrates shared genetic regulation effects across multiple tissues and tissue-specific genetic regulation effects, providing prediction models for each tissue. While AD symptoms are primarily linked to hippocampal and frontal cortex lesions, brain amyloid plaques and atrophy can occur throughout the brain in AD patients (Khan et al., 2020). Furthermore, there has been increased attention on the use of blood biomarkers for AD diagnosis and early screening (Blennow and Zetterberg, 2018). Consequently, we downloaded the corresponding JTI pre-training model and performed TWAS analysis to identify AD risk genes in 13 brain regions and blood, respectively. After removing the duplicate genes, a total of 670 potential genes (FDR < 0.05, Supplementary Table 1) were obtained from 13 brain regions and blood. Since JTI has finally established a relationship between gene expression and AD, it is speculated that the expression pattern of these risk genes is associated with the genetic risk of AD.

**FIGURE 2:**
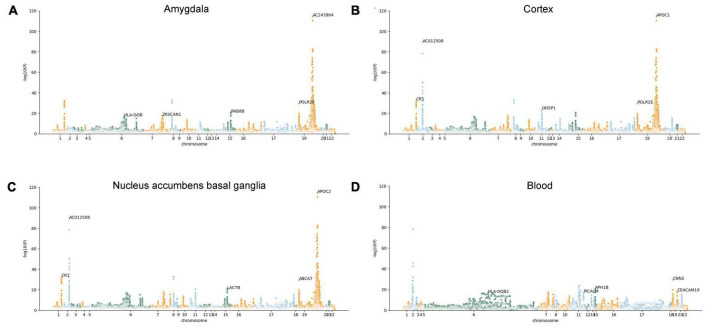
Manhattan plots illustrating MR-JTI results in different brain regions and blood. The vertical axis is the corresponding “-log (*P*-value)” of each gene in the JTI result; the higher the corresponding value of the gene point, the higher the association between the gene and AD. Brain amygdala **(A)**; brain cortex **(B)**, brain nucleus accumbens basal ganglia **(C)**, and blood **(D)**.

However, we are uncertain whether the expression changes of these AD-associated risk factors are the cause or outcome of the disease. Therefore, the MR-JTI analysis was conducted on the genes identified by JTI, integrating LD score, eQTL data, and GWAS summary statistic data. After removing the duplicate genes, 415 AD potential causal risk genes (FDR < 0.05, Supplementary Table 2) were identified from 13 brain regions and blood (Supplementary Table 1). The Manhattan plot illustrates the AD-associated risk factors in 13 brain regions and blood screened by MR-JTI (Figure 2 and Supplementary Figure 1). Among the top five genes in the brain amygdala region, RAB8B and HLA-DOB have been confirmed to be involved in the pathogenesis of AD (Figure 2A; Patel et al., 2021; Martinez et al., 2023). Strong evidences indicates that abnormalities of CR1, APOC1, APOC2, LACTB, and ABCA7 were closely related to AD (Figures 2B, C; Karch and Goate, 2015; Shao et al., 2018; De Roeck et al., 2019; Kulminski et al., 2022; Yu et al., 2022). It is worth noting that the top five genes in the blood have been confirmed to be associated with AD, including HAL-DQB2, PLCALM, APH1B, CNN2, and CEACAM19 (Figure 2D; Logue et al., 2018; Seripa et al., 2018; Gockley et al., 2021; Park et al., 2021; Zhang et al., 2022b).

### Enrichment analysis of AD-associated genes

**FIGURE 3:**
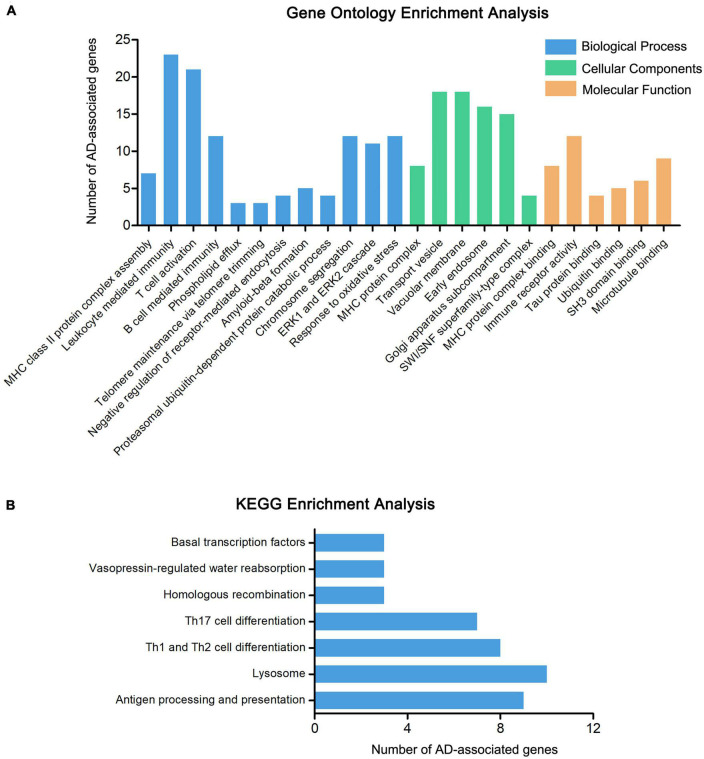
Enrichment analysis of AD-associated genes. **(A)** The GO enrichment analysis of 415 AD-associated genes, including molecular biological process (BP), cellular components (CC), and function (MF). **(B)** The KEGG enrichment analysis of 415 AD-associated genes.

To further explore the relationship between the 415 genes and AD, the pathway and functional enrichment analysis was performed by GO and KEGG. The GO results revealed that the 415 genes were enriched in immune-related pathways (MHC class II protein complex assembly, leukocyte mediated immunity, and T cell activation), ERK1 and ERK2 cascade, Tau protein binding, and Ubiquitin binding, especially enriched in amyloid-beta formation (including genes CLU, APH1B, ABCA7, BIN1, and PICALM) (Figure 3A and Supplementary Table 3). The results of KEGG functional enrichment analysis showed that the AD-associated genes enriched in antigen processing and presentation, lysosome, and Th1 and Th2 cell differentiation (Figure 3B and Supplementary Table 4).

### Fisher test for AD-associated genes and DEGs

**FIGURE 4:**
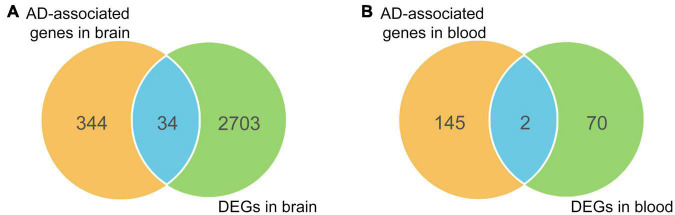
Overlapping differentially expressed genes (DEGs) with causal genes in brain regions **(A)** and blood **(B)**.

To enhance the reliability of screening AD-associated risk factors, we conducted a Fisher test on 415 AD-associated genes using differentially expressed genes (DEGs) in AD. Firstly, we searched the GEO database and analyzed the DEGs in 11 AD-related datasets, comprising eight different brain region datasets and three peripheral blood datasets (Table 1). A total of 2737 unique DEGs (*P* < 0.05 and | log_2_FC| > 1) were identified from eight AD-related GEO datasets of brain regions (Supplementary Table 5), and 147 unique DEGs (*P* < 0.05 and | log_2_FC| > 1) were screened from three peripheral blood GEO datasets (Supplementary Table 5). Venn analysis of 378 AD-associated genes in the brain regions and 2737 DEGs in the brain regions showed that there were 34 common highly reliable genes associated with AD (Figure 4A and Supplementary Table 6). These genes contain APOC1, CR1, CISD1, and others. Additionally, there were two common highly reliable genes associated with AD that were shared between the 72 AD-associated genes in the blood and the 147 DEGs in the blood (Figure 4B), including LAT2 and NDUFS2. Their roles in AD pathology are listed in Table 2.

**TABLE 2:** The details of highly reliable common genes in AD.

Gene symbol	Full name	Function in AD	References
APOC1	Apolipoprotein C1	APOC1 gene polymorphisms associated with AD risk.	Kulminski et al., 2022
APOC2	Apolipoprotein C2	TOMM40-APOE-APOC2 regulatory region methylation status associated with AD.	Shao et al., 2018
ASPHD1*	Aspartate beta-hydroxylase domain containing 1	N/A	
AZGP1	Alpha-2-glycoprotein 1, zinc-binding	Proteomics analysis of 2D-DIGE and iTRAQ labeling technology showed that AZGP1 was differentially expressed in blood serums of AD.	Shen et al., 2017; Zhang et al., 2022a
BEST3*	Bestrophin 3	N/A	
C1QTNF4*	C1q And TNF Related 4	N/A	
CISD1	CDGSH Iron sulfur domain 1	Mitochondrial dysfunction plays an important part in the pathology of several diseases, including AD. The mitochondrial protein CISD1 has emerged as the mitochondrial target of thiazolidinedione drugs such as the antidiabetic pioglitazone.	Geldenhuys et al., 2014
CNTNAP1	Contactin associated protein 1	CNTNAP1 associates with γ-secretase in detergent-resistant membranes and affect Aβ from its precursor protein processing. It may be a potential therapeutic target for AD.	Hur et al., 2012
CR1	Complement C3b/C4b receptor 1	CR1 is one of the most important risk genes for late-onset AD, it plays multiple roles in the onset of AD, such as Aβ clearance, neuroinflammation, and tauopathy.	Zhu et al., 2015
CTSH	Cathepsin H	CTSH highly expression in AD patients and AD animal models and CTSH knockout significantly increased phagocytosis of Aβ peptides.	Li et al., 2023
DNA2*	DNA replication helicase/nuclease 2	N/A	
DNAH11*	Dynein Axonemal heavy chain 11	N/A	
DOC2A	Double C2 domain alpha	DOC2A may be one of predictive biomarkers for AS	Zhou et al., 2022
ERBB2	Erb-B2 receptor tyrosine kinase 2	ErbB2 modulates the proteostasis of APP-CTFs in AD by regulating autophagic flux and it is expected to be a potential therapeutic target for AD.	Wang et al., 2017
ERC2	ELKS/RAB6-interacting/CAST family member 2	ERC2 was upregulated expression in AD patient and mice model.	Lyons et al., 2022
GPR17	G protein-coupled receptor 17	Inhibition of GPR17 with cangrelor ameliorates cognitive impairment and synaptic deficits induced by Aβ1-42 in mice.	Jin et al., 2021
HLA-DQB1	Major histocompatibility complex, class II, DQ Beta 1	HLA-DQB1 gene polymorphism associated with AD risk.	Mansouri et al., 2015
HLA-DRA	Major histocompatibility complex, class II, DR alpha	HLA-DRA was highly expression in AD pathological brain samples, which may contribute the increased intracranial inflammation in AD.	Yokoyama et al., 2016
HLA-DRB1	Major histocompatibility complex, class II, DR beta 1	HLA-DRB1 gene polymorphism associated with AD risk.	Lu et al., 2017
IBSP*	Integrin binding sialoprotein	N/A	
IL34	Interleukin 34	IL-34 injures the formation of macrophages and reduces their ability to uptake pathological forms of Aβ.	Zuroff et al., 2020
INO80E*	INO80 complex subunit E	N/A	
IQCK	IQ motif containing K	IQCK were increased expression in AD brains and amyloid plaques, and it may play a pathogenic role in either Aβ generation or amyloid plaque deposition in AD.	Wang et al., 2022
KCNC2*	Potassium voltage-gated channel subfamily C member 2	N/A	
MAPT-AS1*	MAPT antisense RNA 1	N/A	
NSF	N-Ethylmaleimide sensitive factor, vesicle fusing ATPase	Tau interacts with and dose-dependently reduces the activity of NSF, thus affecting memory formation.	Prikas et al., 2022
OARD1*	O-Acyl-ADP-ribose deacylase 1	N/A	
PRSS35*	Serine protease 35	N/A	
RAB8B	Ras-related protein Rab-8B	Rab8b may be involved in the pathogenesis of AD by affecting autophagy maturation.	Martinez et al., 2023
RGS5*	Regulator Of G protein signaling 5	N/A	
RIN3	Ras and Rab interactor 3	Upregulation of RIN3 induces endosomal dysfunction in AD.	Shen et al., 2020
SEZ6L2*	Seizure related 6 homolog like 2	N/A	
SLC4A8*	Solute carrier family 4 member 8	N/A	
SNX32	Sorting Nexin 32	SNX32 was associated with increased risk of AD.	Kibinge et al., 2020
LAT2	Linker for activation of T cells family member 2	LAT2 may be a key target related to AD immunity.	Li et al., 2022
NDUFS2*	NADH: ubiquinone oxidoreductase core subunit S2	N/A	

*Novel potential AD-related genes identified by MR-JTI and there is no report on the function of this gene in AD.

**FIGURE 5:**
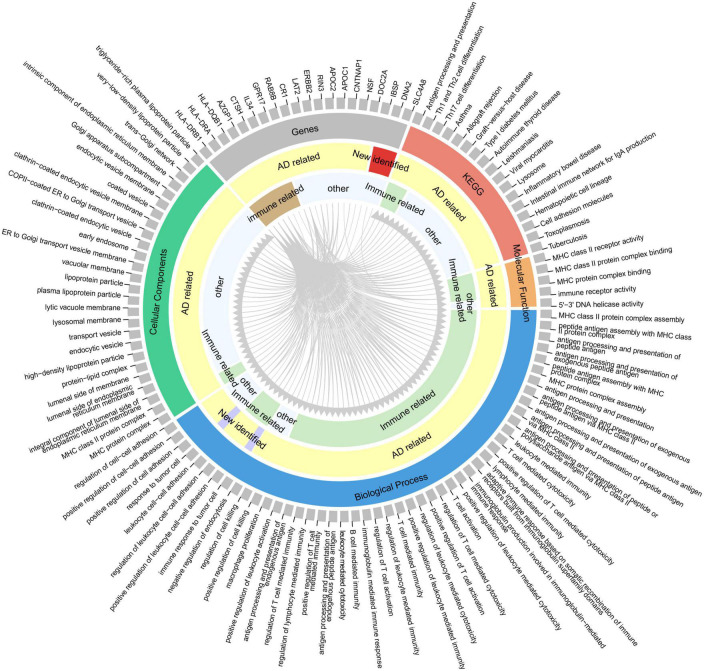
Correspondence between highly reliable genes and enriched pathways. From the outer circle to inner circle, the first circle represents an index containing 20 genes and 95 pathways (enriched by the 415 potential AD-associated genes with adjusted *P* < 0.05); the second circle denotes the gene or pathway type; the third circle is whether the gene/pathway has been reported to be related to AD; the fourth circle indicates whether the gene/pathway is immune-related. Gray lines indicate the correspondence between genes and pathways.

To provide insights for further research on the function of these highly reliable genes in AD, the relationship between the pathways involved in the highly reliable genes and AD was evaluated through manual literature search (Figure 5). The results showed that most of these pathways have either been previously reported or are potentially associated with the pathology of AD, including MHC protein complex assembly, and antigen processing and presentation. Furthermore, it was found that the majority of these pathways were related to immunity. To confirm whether these pathways are indeed immune-related, an additional literature search was conducted.

## Discussion

With the increase in human life expectancy and the intensification of the aging society, the prevalence of AD is also on the rise (Alzheimer’s Association, 2021). However, due to the complex etiology and unknown pathogenesis of AD, effective intervention measures in the clinic are lacking. Additionally, there is a lack of effective targets, creating obstacles for drug discovery. Therefore, we performed TWAS using JTI to identify more potential AD risk factors. A total of 670 AD potential risk genes were identified from 13 brain regions and blood, and further causal inference with MR-JTI identified 415 AD-associated risk genes. Currently, the mainstream view is that one of the causes of AD is the deposition of β-amyloid protein (Aβ) and Tau protein resulting in the death of massive neurons (Masters and Selkoe, 2012). These 415 risk genes also enriched in related pathways, including amyloid-beta formation and Tau protein binding. These pathways involve CLU, APH1B, ABCA7, BIN1, PICALM, and MARK4, all of which have been widely proven to be closely related to the pathogenesis of AD by GWAS and numbers experiments (Jansen et al., 2019; Nott et al., 2019; Li et al., 2021; Schwartzentruber et al., 2021). For example, ABCA7 may stimulate cholesterol efflux from cells into lipoprotein particles and further inhibiting/clearing Aβ aggregates and influencing the risk of AD (Chan et al., 2008; Wildsmith et al., 2013). Phosphorylated MARK4 was highly expressed in AD brain tissue, and it could phosphorylate tau at Ser^262/356^, contributing to tau accumulation, toxicity, and subsequent neurodegeneration (Oba et al., 2020; Waseem et al., 2021). Notably, many genes enriched in immune-related pathways, mainly involving the members of the histocompatibility Complex, Class II, such as HLA-DQA2, HLA-DOB and HLA-DRB1 (Supplementary Tables 3, 4). Some of them have been proven to be contributed to the onset of AD, such as HLA-DRB1, HLA-DQA1, and HLA-DQB1 (McGeer et al., 1988; Payton et al., 2006; Mansouri et al., 2015; Zhang et al., 2022b).

To obtain more reliable AD risk factors, the DEGs in AD were used to conduct the Fisher test on 415 AD-associated risk genes. The 34 common genes were obtained in the brain regions, and two common genes in the blood. The deposition of β-amyloid protein in the brain plays a crucial role in the pathogenesis of AD. Generally, under normal physiological conditions, the production and clearance of Aβ maintain a dynamic balance. However, under pathological conditions, Aβ production increases or clearance decreases, disrupting the balance and leading to excessive deposition of Aβ in the brain. This, in turn, triggers a series of pathological processes, such as mitochondrial dysfunction, oxidative stress, and neurofibrillary tangles (Selkoe, 1993). Here, several risk factors that affect Aβ deposition and formation were also identified, including CNTNAP1, CR1, CTSH, and IL34 (Table 2). CR1, encoding a type-I transmembrane glycoprotein, is one of the most important risk genes for late-onset AD, playing multiple roles in the onset of AD, such as Aβ clearance, neuroinflammation, and tauopathy (Zhu et al., 2015). Additionally, autophagy disorders were proved to be associated with AD, and our study also identified autophagy-related genes, including ERRB2 and RAB8B, which have been preliminarily confirmed to be associated with the pathogenesis of AD (Wang et al., 2017; Martinez et al., 2023). Growing evidence from clinical and pathological studies indicates the important relationships between the ongoing deterioration of brain cholesterol metabolic disturbance and AD pathophysiology (Vestergaard et al., 2010). APOC1 and APOC2, both belonging to the apolipoprotein family, and their gene polymorphisms have been reported to be associating with the onset of AD by many studies (Cervantes et al., 2011; Kulminski et al., 2022). In summary, 21 out of 36 highly reliable genes can participate in the pathology of AD through various mechanisms.

More importantly, we have identified many new AD risk factors (Table 2), and their specific involvement in AD has not been clearly reported. By analyzing the relationship between highly reliable genes and enrichment pathways, clues were provided for future research on the functions of these newly identified genes in AD (Figure 5). Most of these pathways have been either been reported or potentially associated with the pathology of AD, including multiple innate and adaptive immune pathways, cell death regulation, DNA repair, cell adhesion, lipoprotein metabolism, protein endocytosis and exocytosis. Therefore, these pathways deserve special attention in future AD research. The newly identified AD risk genes may participate in the occurrence and development of AD through these pathways. For instance, several studies have reported abnormal structure and function of mitochondria in the AD brain, leading to abnormal energy metabolism (Kerr et al., 2017). This abnormal energy metabolism can impact the synaptic plasticity of neurons, thereby affecting memory and learning. CISD1 and DNA2 were reported to be related to mitochondrial function (Geldenhuys et al., 2014; Ding and Liu, 2015), but further detailed and in-depth research is needed to understand their involvement in AD. This implies that significant efforts are still required to explore and investigate the risk factors of AD.

Although many AD-associated targets have been identified by GWAS, high-throughput sequencing, molecular epidemiology, and other methods in the past few decades, it is still a drop in the bucket to improve the awareness of the pathogenesis of AD and the diagnosis and treatment of AD. In this study, we utilized the improved TWAS method, MR-JTI, to integrate and analyze LD score, GTEx eQTL data, and GWAS summary statistic data. A total of 415 AD-associated genes were identified, and the 36 more reliable AD risk was further confirmed by using 11 AD-associated datasets and the Fisher test. The identification of these genes is not only the verification of reported AD-associated genes but also provides new potential AD biomarkers for follow-up research.

## Data availability statement

The raw data used in this study come from public databases, and the link and accession numbers are listed in the text. The resulting data presented in this study are included in the article/Supplementary material.

## Author contributions

YW and YT: conceptualization. YW, PL, T-HL, and YT: methodology. PL, XL, YH, and LH: data collection. YW, PL, YH, and YT: data analysis. YW, PL, XL, and YT: data curation. YW, PL, XL, and LH: visualization. YW, PL, AG, T-HL, and YT: drafted the manuscript. All authors contributed to the article and approved the submitted version.

## Footnotes

1
https://www.ebi.ac.uk/gwas/

2
https://www.gtexportal.org/home/

3
https://zenodo.org/record/3842289#.Y9iX_XZByyx

4
https://ctg.cncr.nl/software/MAGMA/ref_data/

5
http://www.ncbi.nlm.nih.gov/geo

6
https://david.ncifcrf.gov/tools.jsp

7
https://www.genome.jp/kegg/

8
http://geneontology.org

9
https://www.innatedb.com/

10
https://www.immport.org/home
