# Supplementary material for: Identification of highly reliable risk genes for Alzheimer’s disease through joint-tissue integrative analysis
Source: Front Aging Neurosci. 2023 Jun 21;15:1183119. doi: 10.3389/fnagi.2023.1183119 (PMC10320295; doi:10.3389/fnagi.2023.1183119)
Supplement: Supplementary file 2 [file Data_Sheet_1.DOCX]

**Identification of highly reliable risk genes for Alzheimer's disease through joint‑tissue integrative analysis**

Yong Heng Wang^1,2 ¶^, Pan Pan Luo^1 ¶^, Ao Yi Geng^1^, Xinwei Li^3^, Tai-Hang Liu^1,2^, Yi Jie He^1^, Lin Huang^1^, Ya Qin Tang^1, *^

^1^ Department of Bioinformatics, School of Basic Medical Sciences, Chongqing Medical University, No.1 Yixueyuan Road, Yuzhong District of Chongqing 400016, China.

^2^ Joint International Research Laboratory of Reproduction & Development, Chongqing Medical University, No.1 Yixueyuan Road, Yuzhong District of Chongqing 400016, China.

^3^ School of microelectronics and communication engineering, Chongqing University, No.174 Shazheng Street, Shapingba District of Chongqing 400044, China.

**Correspondence:** Box 197, Chongqing Medical University, No.1 Yixueyuan Rd, Chongqing, 400016, PR China. Tel.: +86 023 68485868. * Ya Qin Tang (yqtang@cqmu.edu.cn).

¶ Y.H. Wang and P.P. Luo contributed equally to this work.


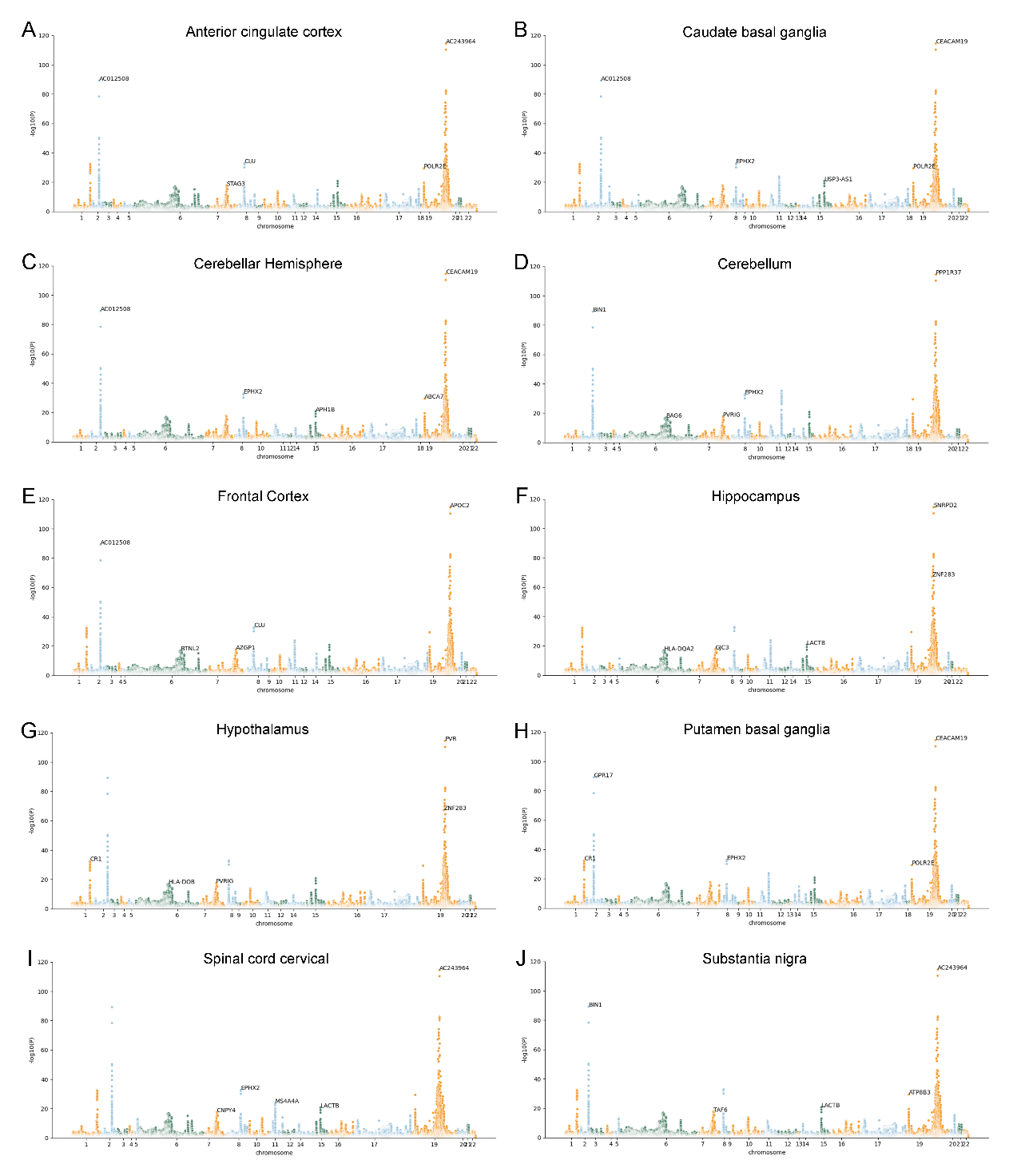


**Supplementary Figure 1. Manhattan plots of the MR-JTI results in different brain regions.**
